# Induced Pluripotent Stem Cell Derived Macrophages as a Cellular System to Study *Salmonella* and Other Pathogens

**DOI:** 10.1371/journal.pone.0124307

**Published:** 2015-05-06

**Authors:** Christine Hale, Amy Yeung, David Goulding, Derek Pickard, Kaur Alasoo, Fiona Powrie, Gordon Dougan, Subhankar Mukhopadhyay

**Affiliations:** 1 Wellcome Trust Sanger Institute, Wellcome Trust Genome Campus, Hinxton, Cambridge, United Kingdom; 2 Kennedy Institute of Rheumatology, University of Oxford, Oxford, United Kingdom; University of Kansas Medical Center, UNITED STATES

## Abstract

A number of pathogens, including several human-restricted organisms, persist and replicate within macrophages (Mφs) as a key step in pathogenesis. The mechanisms underpinning such host-restricted intracellular adaptations are poorly understood, in part, due to a lack of appropriate model systems. Here we explore the potential of human induced pluripotent stem cell derived macrophages (iPSDMs) to study such pathogen interactions. We show iPSDMs express a panel of established Mφ-specific markers, produce cytokines, and polarise into classical and alternative activation states in response to IFN-γ and IL-4 stimulation, respectively. iPSDMs also efficiently phagocytosed inactivated bacterial particles as well as live *Salmonella* Typhi and *S*. Typhimurium and were able to kill these pathogens. We conclude that iPSDMs can support productive Salmonella infection and propose this as a flexible system to study host/pathogen interactions. Furthermore, iPSDMs can provide a flexible and practical cellular platform for assessing host responses in multiple genetic backgrounds.

## Introduction

Macrophages (Mϕs) are an important line of defence against many pathogens, being involved in microbial recognition, phagocytosis, killing, secretion of inflammatory mediators as well as initiation of adaptive immune responses [1]. However, a number of pathogens have developed specific evasion strategies to hijack the killing machinery and preferentially infect Mϕs to avoid immune effectors including antibodies [2,3].

*Salmonella* have evolved a variety of genetic systems to facilitate controlled entry into and survival within Mϕs as a general infection-associated lifestyle [4–6]. Immortalised human Mϕ-like cell lines have been widely used to explore the biology of such interactions in part because primary Mϕs are difficult to obtain in reproducible numbers, show significant levels of donor variability and are relatively resistant to genetic manipulation [7]. Thus, there is a need to develop new genetically tractable cellular models in this area.

Recently methods have been developed to differentiate Mϕ-like cells from human induced pluripotent stem cells (iPSC) [8,9]. These iPSC-derived Mϕs (iPSDM) are genetically highly related to their original donor cells, share striking phenotypic and functional similarities with primary human Mϕs and are amenable to genetic manipulation. Previous studies utilised iPSDMs to model rare genetic defects that impact Mϕ functions [10–12]; or investigated their utility in cellular therapies [13–15]. However, the potential of iPSDMs in host-pathogen interaction studies, especially to study human adapted pathogens, has not been fully evaluated.

Consequently, in this study, we exploit *Salmonella enterica serovar Typhimurium* (*S*. Typhimurium) and *S*. Typhi to infect iPSDMs and provide a detailed methodology for this approach. We also compare the properties of infected iPSDMs with the human monocyte-like cell line THP-1, frequently used to characterise *Salmonella* cellular interactions and report these comparative data.

## Materials and Methods

### Maintenance of iPSCs and directed differentiation into mature Mϕs

Human dermal fibroblast derived iPSC line CRL-1 has been described before; and it was a kind gift from Dr Ludovic Vallier [16]. Undifferentiated CRL-1 was maintained on a monolayer of mitotically inactivated mouse embryonic feeder (MEF) cells in Advanced Dulbecco’s modified Eagles/F12 medium (DMEM/F12), supplemented with 20% Knockout replacement serum (KSR), 2mM L-Glutamine, β-mercaptoethanol (0.055 mM) and 8 ng/ml recombinant human FGF2 (RnD system); as described previously [9]. These cells were differentiated into Mϕs following a previously published method [9]; and any specific modification of this protocol is discussed in detail within the result section. Briefly, this protocol involves key stages of differentiation- i) formation of 3 germ layer containing embryoid bodies (EBs) from iPSCs on withdrawing FGF, ii) long term production of myeloid precursor cells from EBs in presence of 25ng/ml IL-3 and 50ng/ml M-CSF (both RnD) and iii) terminal differentiation and maturation of myeloid precursors into matured Mϕs in the presence of higher concentrations of M-CSF (100ng/ml).

### Culture of THP-1 cells and their maturation into Mϕs

The human monocyte-like cell line THP-1 was obtained from ECACC (#88081201) and routinely cultured in RPM1 1640 supplemented with 2mM L-Glutamine and 10% heat-inactivated Foetal calf serum (FCS). Cells were differentiated into mature Mϕ-like cells by stimulating with 100ng/ml Phorbol 12-Myristate 13-acetate (PMA) for 3 days and replaced with medium without PMA for 1 day prior to assay [17].

### RNA extraction and sequencing

RNA was extracted from iPS cells or from iPSDMs with RNeasy Mini Kit (Qiagen) according to manufacturer’s protocol. After extraction, the sample was incubated with Turbo DNase at 37°C for 30 minutes and subsequently re-purified using RNeasy clean-up protocol. Standard Illumina single-stranded poly-A enriched libraries were prepared and then sequenced 5-plex on Illumina HiSeq 2500 (2 x 75bp paired-end) generating 20–50 million paired-end reads per sample. RNA-Seq data was analysed using standard methodologies.

### Cellular phenotyping by Flow Cytometry

iPSDM and THP-1 Mϕs were grown on tissue culture plastic dishes using RPMI media. In some experiments cells were prestimulated overnight with either 20ng/ml recombinant human IFN-γ or 50ng/ml recombinant human IL-4. Cells were detached using Lidocaine solution as described before [18]. Mϕs or immature myeloid precursors were plated into 96 well round bottom plates at a density of 10^5^ cells/well and incubated for 30 minutes at 4°C in 100μl of FACS blocking buffer containing 5% FCS in phosphate buffered saline (PBS), 0.1% sodium azide and 2μl of Trustain Fc block. 5μl of directly conjugated anti-human antibodies against individual Mϕ plasma membrane antigens (CD14 AF488, CD16 APC-Cy7, CD44 PerCpCy5.5, CD64 AF700, CD200 APC (AbD Serotec), CD206 APC, CD54 PE, CD11c APC, CD163 PE, HLA DP-DQ-DR AF488 and CD86 Horizon v450 from Becton Dickinson) or appropriate isotype matched control antibodies were added to each well and incubated for a further 30 minutes. Cells were washed twice with FACs buffer, resuspended in PBS and analysed on a Becton Dickinson FACsAria11 using FACS Diva software or Flowjo. Intracellular antigen CD68 was detected after fixation and permeabilisation using Beckton Dickinson’s Cytofix/Cytoperm kit as per manufacturer’s instruction.

### Mϕ stimulation with toll-like receptor agonists

iPSDM and THP-1 Mϕs were seeded into wells of 24 well plates at 3x10^4^/well in RPM1 1640 Mϕ medium and allowed to settle for 24 hours. Cells were stimulated for indicated time points with the agonist of human toll-like receptor 3 (TLR-3) Poly IC (1μg/ml), TLR-9 agonist CPG ODN2006 (2.5μM), TLR5 agonist Flagellin (1μg/ml), TLR-1/2 agonist PAM3CS4 (100ng/ml) and TLR-4 agonist LPS (1 ng/ml). Supernatants were harvested, filtered through 0.2uM filters and stored in -80C before cytokine analysis.

### Phagocytosis assays

Approximately 3x10^4^ iPSDMs or differentiated THP-1 Mϕs were seeded, on acetone washed sterile 13mm diameter coverslips placed inside the 24 well tissue culture plate, using RPMI 1640 Mϕ media and allowed to settle for 24 hours and incubated with reconstituted Rhodo-red bioparticles or GFP expressing *S*. Typhimurium SL1344 or *S*. Typhi BRD948 [19] at a multiplicity of infection (MOI) ratio of 20:1. After indicated time points, cells were washed twice with D-PBS and fixed with 4% formaldehyde for 20 minutes. In some experiments cells were permeabilised and immunostained for *Salmonella* common surface antigen (CSA); and analysed with a Zeiss LSM 510 Meta confocal microscope.

### Anti-human cytokine/chemokine multiplex bead assays

25μl of Mϕ culture supernatants were analysed for cytokine/chemokine concentrations. Millipore customised anti-human Milliplex magnetic bead kits were utilised as per manufacturers instruction and a selection of the analytes IFN-γ, GM-CSF, TNF-α, IL-1β, IL-2, IL-4, IL-5, IL-6, IL-7, IL-8, IL-10, IL-12p70 or IL-13 utilised. Data was acquired on a Luminex FlexMap3D and analysed with either Luminex or Masterplex QT software.

### Bacteria and growth conditions

*S*. Typhimurium SL1344 and *S*. Typhi BRD948 both harbouring the reporter plasmid pssaG::GFP were grown on L-broth or L-agar containing ampicillin at 100μg/ml final concentration. Culture media was supplemented with aromatic amino acid components as described previously when growing *S*. Typhi BRD948 [19]. *S*. Typhimurium SL1344(pssaG::GFP) has been described previously [20]. Briefly, the promoter region of *ssaG* was cloned into plasmid pQF50 [21], upstream of a promoterless GFP gene derived from pmutGFP3.1 (Promega labs, USA). Growth in conditions favouring the activation of Salmonella Pathogenicity Island-2 (SPI-2), of which pssaG is a component leads to expression of GFP via the ssaG promoter region. For the infection studies we grew the cultures up statically overnight to simulate microaerophilic conditions at 37°C. The culture OD at 600nm was measured and the colony forming units (cfu)/ml calculated. An MOI of 20:1 was used for all infections (bacteria:tissue culture cell ratio).

### Gentamicin protection assay

Gentamicin-protection assays were carried out as described previously [22] with minor modifications. Briefly, 2x10^5^ Mϕs were plated on 24 well plates one day prior to assay in antibiotic free media. In some cases cells were stimulated overnight with 20ng/ml IFN-γ. On the day of infection, cells were washed three times with PBS and *S*. Typhimurium and *S*. Typhi were added to the media at indicated MOI and incubated at 37°C for 0.5 hr. After incubation, cells were washed 3 times and incubated for a further 2, 4 or 24 hrs with media containing 50ng/ml Gentamicin to kill extracellular bacteria. After each time point, supernatants were harvested, filtered and stored in -80°C for future analysis of cytokine concentration. Cells were lysed in 1% Triton X-100 in PBS solution and multiple 10-fold serial dilutions were plated on LB agar containing 100μg/ml ampicillin. Aromatic amino acids were added to the *S*. Typhi agar plates. Numbers of Gentamicin-resistant intracellular bacteria were determined by counting colonies the next day.

In initial experiments we assessed macrophage viability after Salmonella infection by Trypan blue exclusion method. We observed some cytotoxicity in longer time points, *S*. Typhimurium is generally more cytotoxic compared to *S*. Typhi, but no significant difference in toxicity was observed between two macrophages (not shown). The time points for subsequent experiments were chosen accordingly to avoid Salmonella induced cytotoxicity.

### Statistical analysis

All experiments were repeated at least three times in triplicate. Data from a representative experiment are presented as mean ± SD. Two tailed Student’s t test was performed using Graph pad Prism software to determine statistical significance and p values ≤0.05 considered as significant.

## Results

### A simple, efficient and scalable differentiation protocol for generating iPSDM

**Fig 1:**
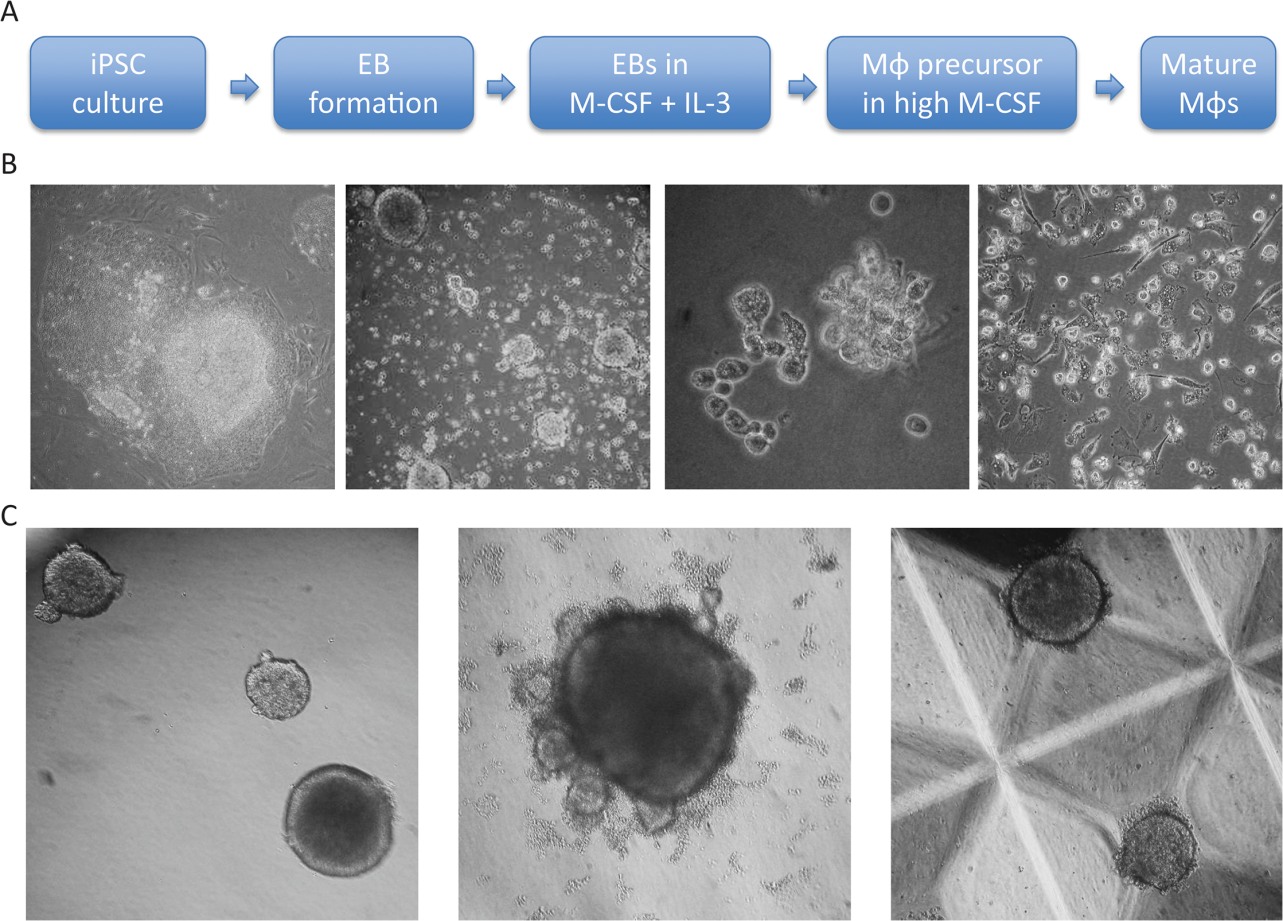
Directed differentiation of Mϕs from human iPSCs. A) A schematic diagram showing specific culture conditions required for each differentiation step from iPSCs to generation of Mϕs; as well as phase contrast photomicrographs of each differentiation step (B). (C)Phase contrast micrographs showing distinct size and morphologies of EB’s generated in 10cm^2^ dish, 96 round bottomed wells and Aggrewell plates.

A number of methods have been developed to differentiate Mϕs from human embryonic stem cells (ESCs) and iPSCs [8,9] and all employ key differentiation steps including- i) maintenance and expansion of iPSCs in culture, ii) formation of embryoid bodies (EB) harbouring three germ layers, iii) Generation of non-adherent myeloid precursor cells from EBs in the presence of the myelogenic cytokines IL-3 and M-CSF and iv) terminal differentiation and maturation of myeloid precursors into matured Mϕs in the presence of a higher concentration of M-CSF. A schematic diagram of individual differentiation steps and their phase contrast photomicrographs are depicted in **Fig 1A** and **1B**. Despite overall similarities, there are subtle variations in published methods; such as iPSCs can be cultured either on a monolayer of feeder cells in the presence of animal serum; or on a layer of extracellular matrix component in a serum-free defined media. Similarly, EBs can be generated utilising either 10cm^2^ dishes, 96 well plates or AggreWells; and myeloid precursors can be generated by culturing EBs in serum free X-vivo media or serum supplemented media. Here we systematically compared the impact of these subtle variations in terms of yield of iPSDMs, and established a relatively simple experimental protocol that generates high number of iPSDMs.

**Fig 2:**
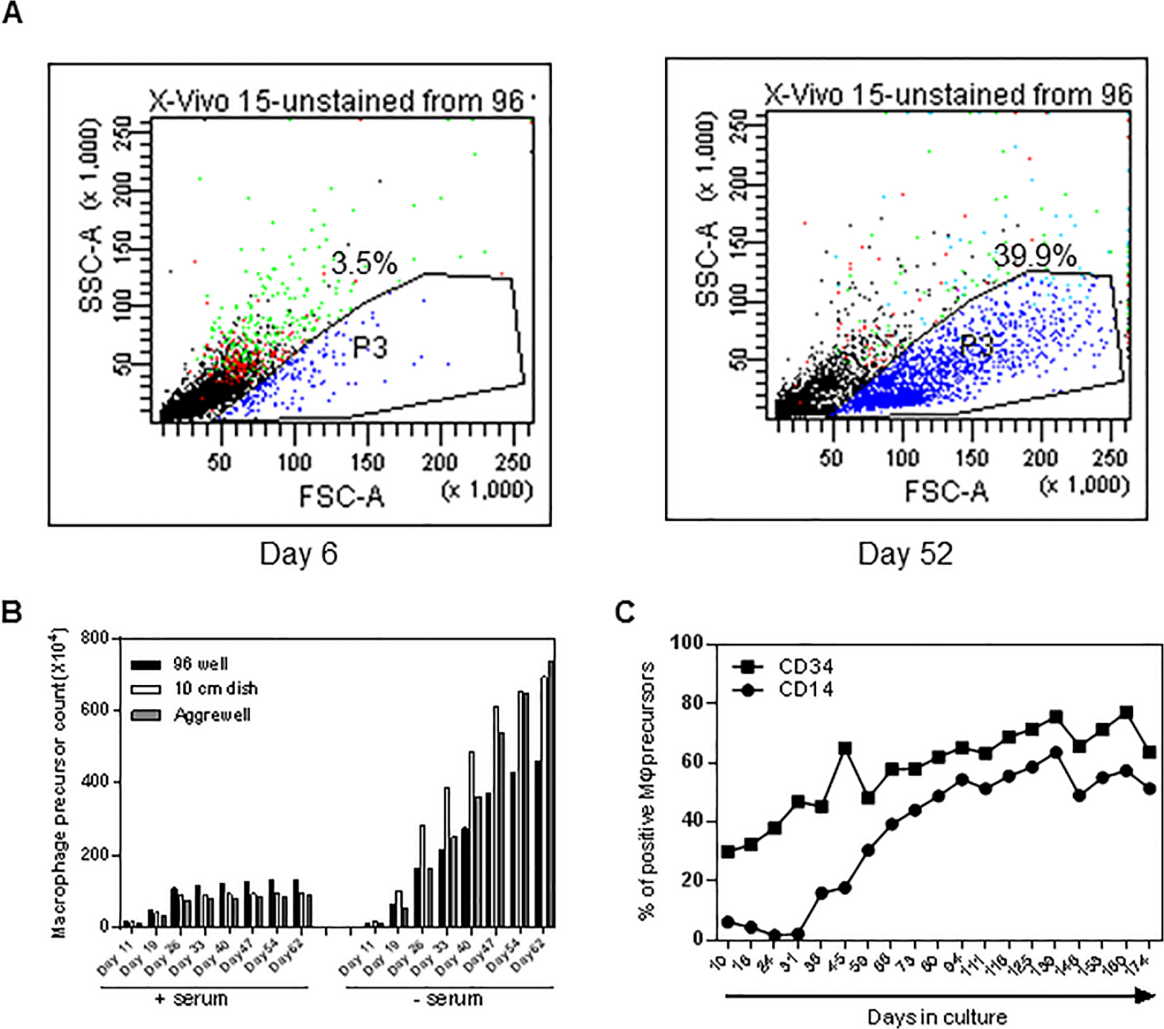
Effects of foetal calf serum on generation of macrophage progenitors. (A) Forward and side scatter of myeloid precursor cells collected at different time points show a time dependent accumulation of larger granular cells (depicted in p3 gate in blue) that correlates with number of matured Mϕs obtained in subsequent differentiation steps. (B) EB’s generated in 10cm^2^ dishes, round bottomed 96 wells or Aggrewell plates were cultured in myeloid differentiation media containing IL-3 and M-CSF in the presence or absence of serum. Numbers of accumulated myeloid precursor cells were counted every week and cumulative numbers over a 2 month period are plotted in the graph. (C) Line diagram showing percentage of myeloid precursor cells that are positive for the haematopoietic lineage marker CD34 and the myeloid lineage marker CD14.

**Fig 3:**
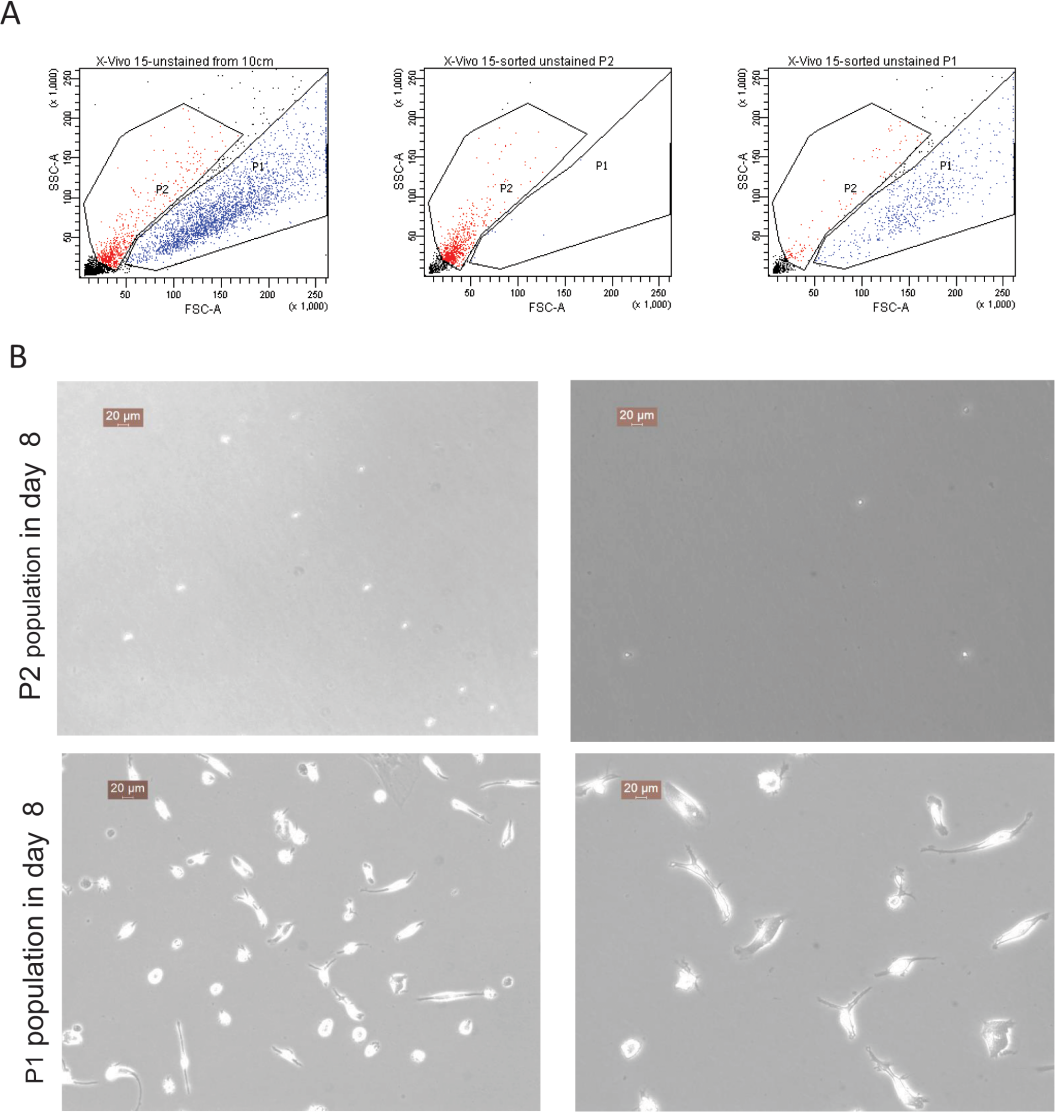
Haematopoitic precursors have distinct potentials for Mϕs generation. (A) Left panel showing forward and side scatters of and large (P1) and small (P2) precursor populations. Middle and right panels are showing purity of P2 and P1 populations respectively, after FACS sorting. (B) Differentiation of Mϕ after culturing sorted populations separately. Left and right panels are showing low and high magnification micrograph respectively.

iPSCs were cultured on a monolayer of inactivated mouse feeder cells in serum containing advanced DMEM/F12 media and EBs were formed in either 10cm^2^ Sterilin bacterial dishes, low adherence 96 wells or in Aggrewell plates respectively **(Fig 1C**). Approximately similar numbers of EBs were divided into two groups and further cultured in either serum free X-vivo media or serum-containing advanced DMEM/F12 media supplemented with IL-3 and M-CSF. Two weeks after transferring the EBs into myeloid differentiation media non-adherent myeloid precursors started accumulating in the media. The numbers of these precursors were counted every week and the expression of the haematopoietic lineage marker CD34 and myeloid lineage marker CD14 were assessed by flow cytometry. We observed that in the initial weeks a relatively homogeneous population of cells accumulated in these cultures that are morphologically smaller (assessed by microscopy and forward and side scatter of FACS), express CD34 but lack CD14. These immature haematopoietic precursors failed to generate any iPSDMs in subsequent culture in the higher M-CSF concentration (data not shown). However, after a few weeks a separate precursor cell population appeared that were larger, had dendrite like structures and expressed both CD34 and CD14 on their surface. The percentage of these larger CD34+CD14+ myeloid precursors was directly correlated with yield of iPSDM **(Fig 2A, 2B and 2C)**. To further characterise these two precursors, we FACS sorted these two populations and placed them separately into macrophage differentiation culture. After 7 days CD14+ larger precursors attached onto tissue culture plastic and developed a characteristic spindle shaped morphology of macrophages, whereas CD34+ smaller precursors did not convert into macrophages, became apoptotic and did not survive in M-CSF containing media (**Fig 3**).Thus, we show that the formation of EBs by different methods has no significant impact on the frequency of myeloid precursors and the subsequent yield of mature Mϕs. However, the presence of serum in myeloid differentiation media significantly compromised the differentiation of myeloid precursors from EBs. Therefore, in all subsequent experiments X-vivo serum free media was used to differentiate EBs into myeloid precursors.

### iPSDMs express lineage specific and maturation markers of primary human Mϕs and can be polarised into classical and alternative activation states

**Fig 4:**
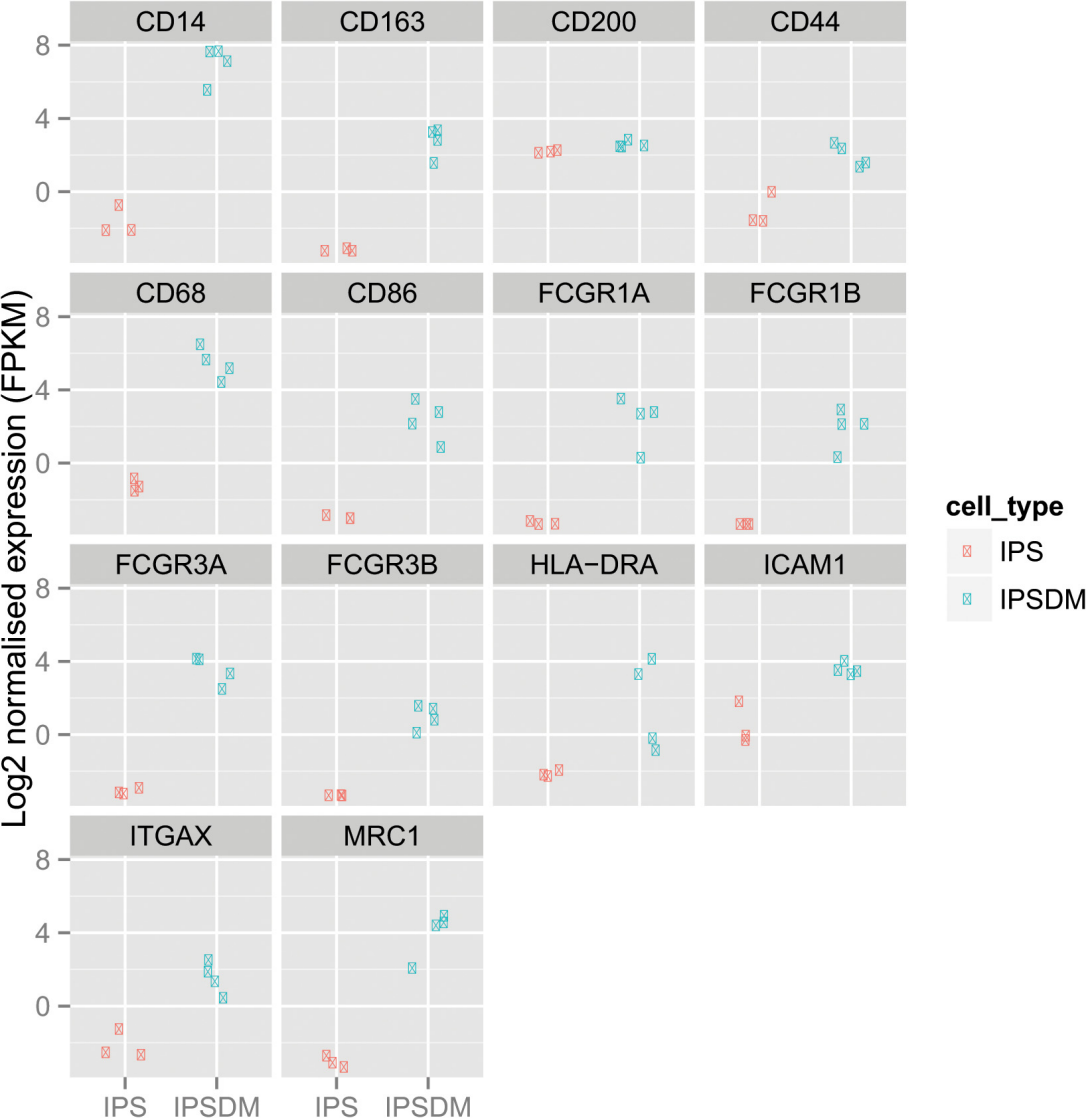
Comparison of mRNA expression of Mϕ-specific markers between undifferentiated iPS cells and iPSDMs. RNA was extracted and sequenced from undifferentiated iPS cells differentiated iPSDMs and log2 normalised expression value for indicated Mϕ surface markers were plotted. Data from three independent replicate is presented.

**Fig 5:**
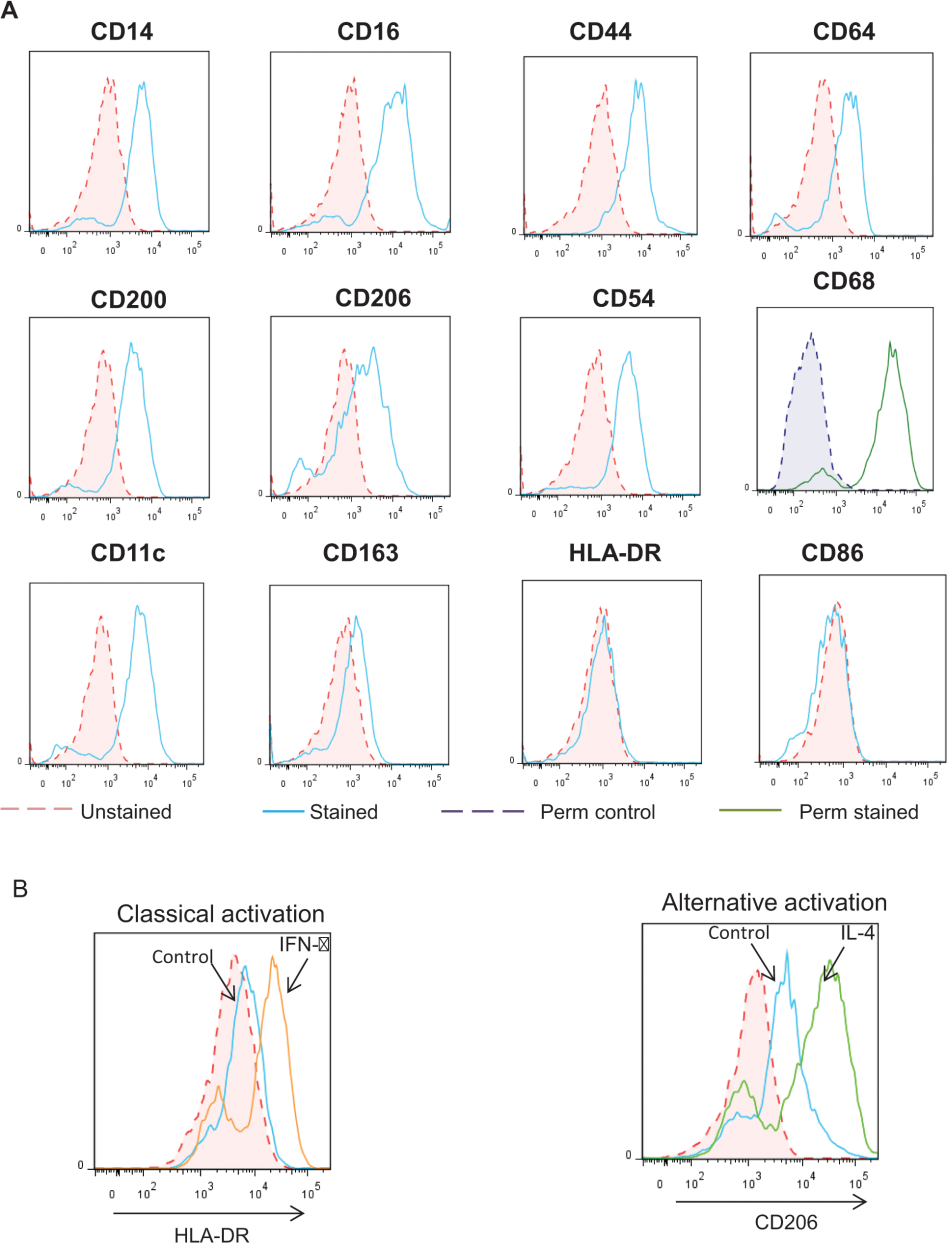
iPSDMs express pan-Mϕ lineage markers in the resting state and induce specific activation markers after classical and alternative activation. A) Histograms showing expression of established markers of resting Mϕs CD14, CD16, CD44, CD64, CD200, CD206, CD54, CD68, CD11c, CD163 and activation markers HLA-DR and CD86 in unstimulated iPS-derived Mϕs. (B) Histogram showing expression of classical activation (M1) marker HLA-DR, DP, DQ and alternative activation marker and CD206 after overnight stimulation with 20ng/ml IFN-γ and 50ng/ml IL-4 respectively.

Terminal differentiation of iPSC derived myeloid precursors in the presence of higher concentrations of M-CSF induced a homogenous looking population of adherent cells that are morphologically similar to mature macrophages. To further assess the phenotypic characteristics of these iPSDMs, global gene expression profiles of undifferentiated iPS cells and iPSDMs were compared by RNASeq and mRNA expression of a range of well-established Mϕmarkers were detected (**Fig 4**). Furthermore, FACS showed that iPSDM express high levels of CD11c, CD14, CD16, CD44, CD64, CD54, CD200R, CD206 and CD68 and to a lesser extent CD163 on their cell surface, indicating that iPSDMs are phenotypically very similar to mature human Mϕs **(Fig 5A)**. Furthermore, these cells did not detectably express activation markers such as HLA-DR and CD86, indicating that the differentiation protocol allows them to fully mature but does not significantly immunologically activate them **(Fig 5A)**. By contrast, treatment with either the T helper cell 1 (Th1) associated cytokine IFN-γ or the (Th2) associated cytokine IL-4 polarise the Mϕs into so-called classical and alternative activation states respectively. Further, the surface expression of HLA-DR and CD206, two established markers of classical and alternative activations were significantly upregulated following IFN-γ and IL-4 treatment respectively (**Fig 5B).**

### Functional comparison between iPSDM and THP-1 Mϕs for phagocytic and secretory response to pathogen components

**Fig 6:**
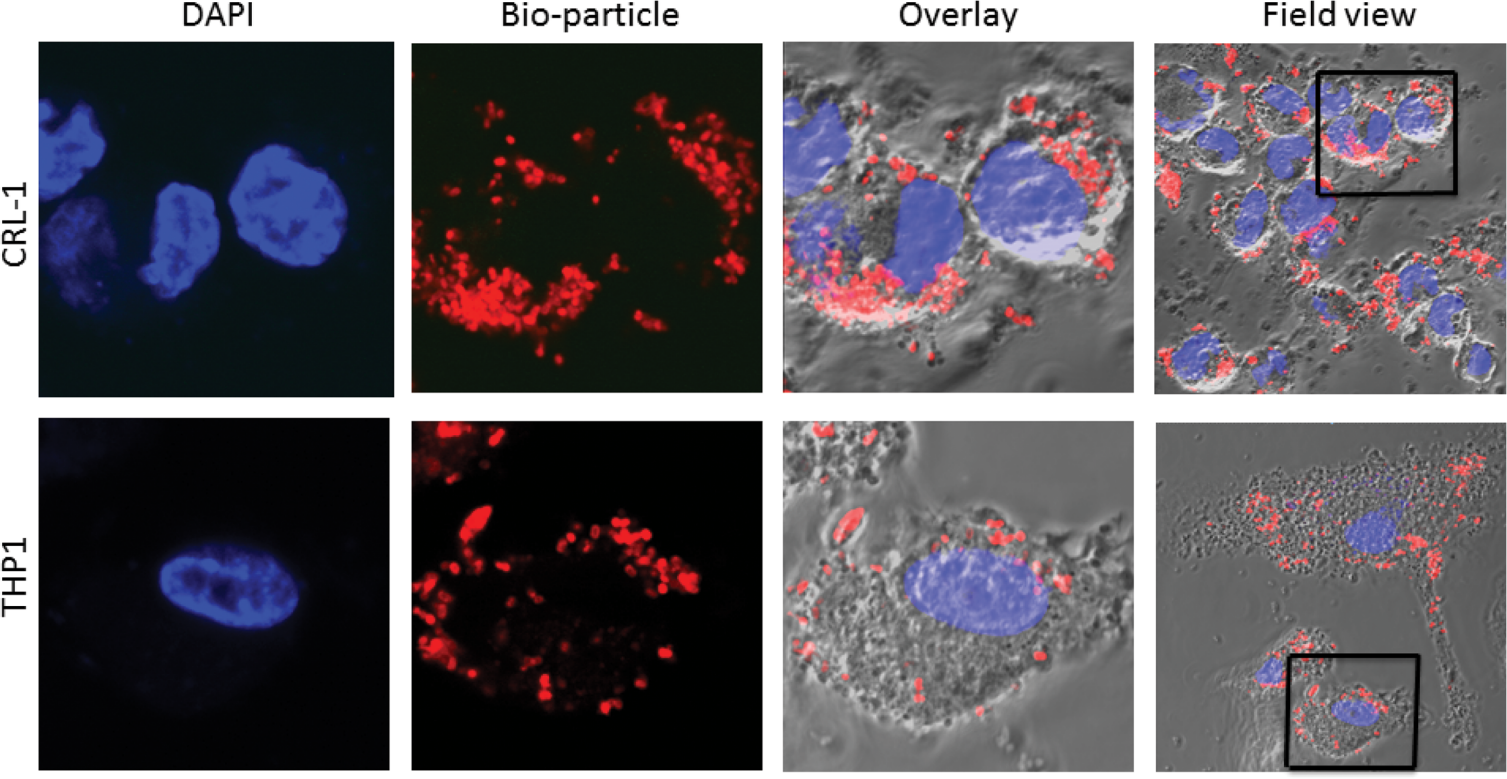
iPSDMs phagocytose inactivated pathogen-like particles. A) Human iPS–derived Mϕ and PMA treated human Mϕ-like cell line THP-1 Mϕs were incubated with Rhodamine labelled inactivated bacteria (Rhodo bioparticles) for 30 minutes and their phagocytic ability assessed by fluorescent microscopy.

**Fig 7:**
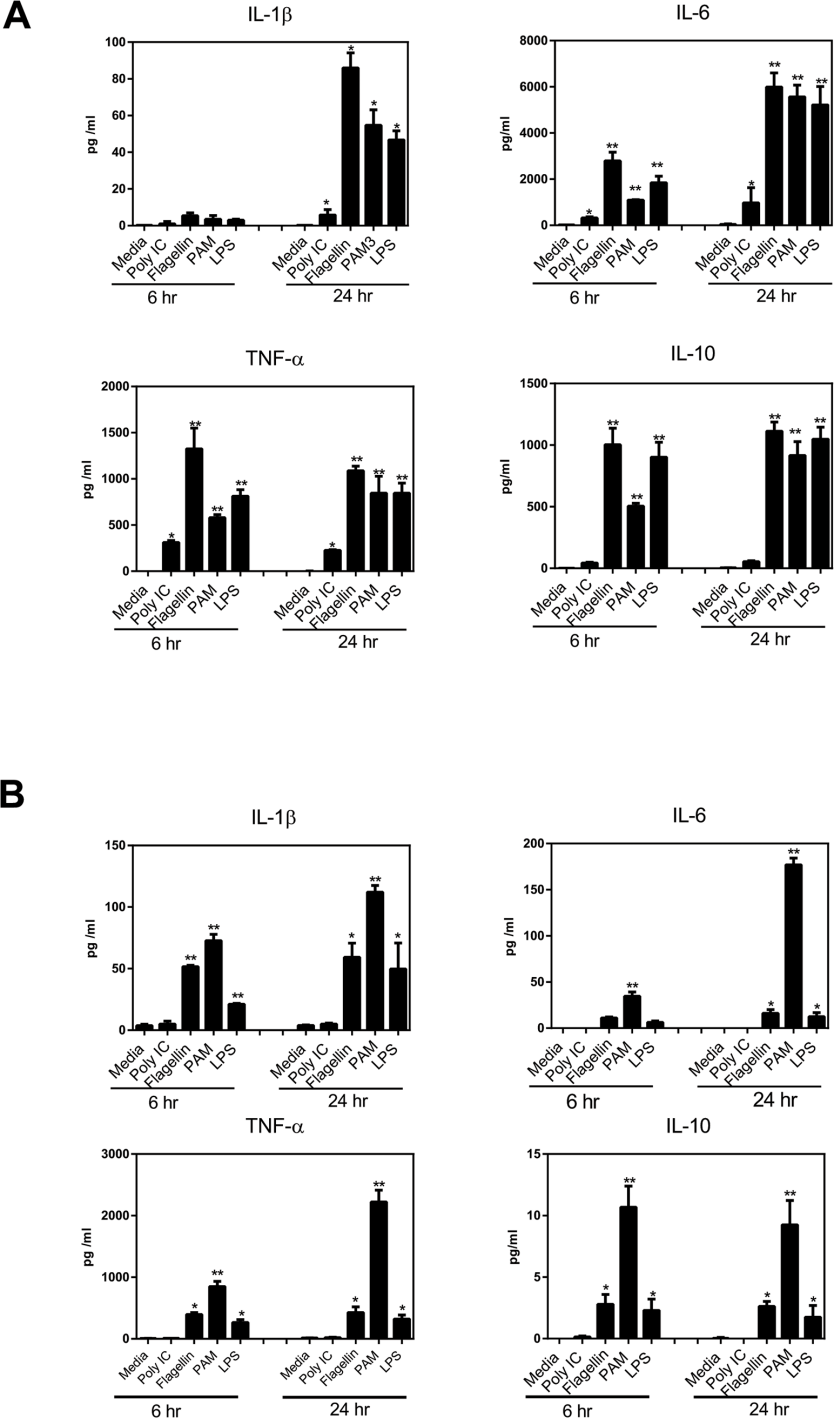
iPSDMs induce inflammatory cytokines in response to purified TLR agonists. iPSDM (A) and THP-1 Mϕs (B) were stimulated with Toll-like receptor agonists Poly IC, Flagellin, PAM3CSK, and LPS for 6 and 24 hrs and secretion of IL-1β, IL-6, TNF-α and IL-10 was determined utilising Millipore Milliplex custom kits on a Luminex FlexMap 3D platform. Representative data from 3 independent experiments are presented as mean ± SD. Statistical significance is determined by comparing with unstimulated cells. P<0.05(*), P<0.01(**) and non-significant (NS) values are indicated within the figure.

Phagocytosis and secretion of cytokines are two fundamental innate immune responses that initiate following macrophage exposure to pathogen components. The phagocytic and secretory responses of iPSDMs were compared with the PMA differentiated human Mϕ-like cell line THP-1. Both iPSDM and THP-1 Mϕs efficiently taken up rhodamine-labelled Rhodo bio-particles and there was no significant difference in kinetics or ability for total particle uptake **(Fig 6)**. Similarly, both cell types secreted the proinflammatory cytokines TNF-α, IL-6 and IL-1β after stimulation with selected TLR agonists **(Fig 7)**. However, we observed that cytokine responses are generally more robust in iPSDMs **(Fig 7A)** compared to THP-1 Mϕs **(Fig 7B)**; the only exception was IL-1β which showed enhanced expression in THP-1.

### iPSDMs take up *Salmonella* and show robust cytokine responses and bacterial killing activity

*S*. Typhimurium and *S*. Typhi are relatively well studied in THP-1 and other Mϕ-like cell lines. To investigate the interactions of isolates of these two serovars with iPSDMs, these and THP-1 Mϕs were generated on glass coverslips and infected with either *S*. Typhimurium SL1344(pssaG::GFP) or *S*. Typhi BRD948(pssaG::GFP) at MOI 20:1 for 0.5hr in antibiotic free media. Extracellular organisms were then killed by further incubation in gentamicin containing media, and after different time points the cells were fixed in 4% formaldehyde, permeabilised and stained using an antibody against Salmonella common surface antigen (CSA). Expression of GFP and CSA were analysed by confocal microscopy where GFP expression reported for intracellular bacteria within a Salmonella containing vacuole (SCV) via activation of pssaG and CSA staining provided an independent measure of bacterial entry.

**Fig 8:**
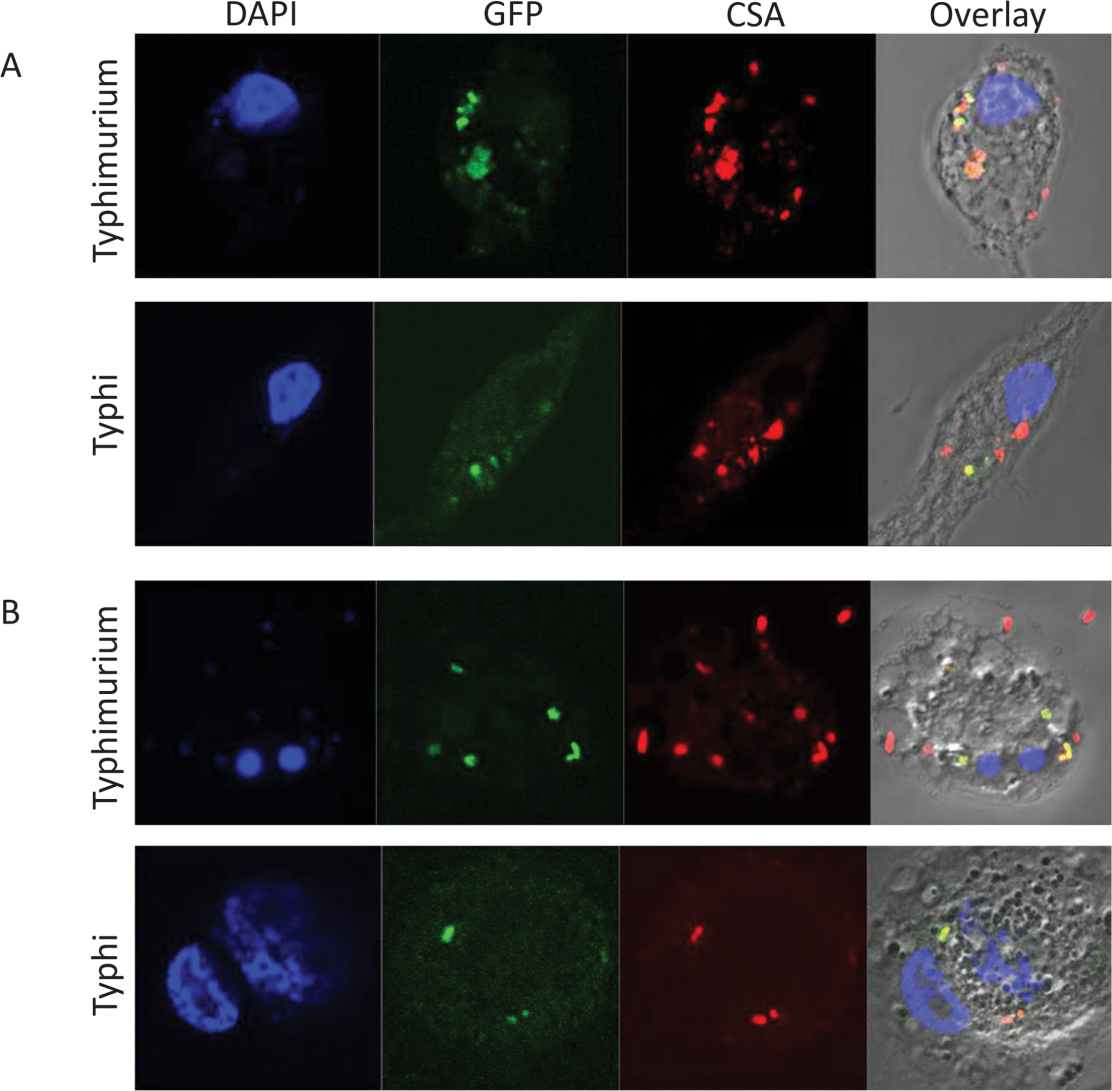
iPSDMs support productive infection of *S*. Typhimurium and *S*. Typhi. **A**) iPSDMs and THP-1 cell lines were infected with GFP expressing *S*. Typhimurium SL1344(pssaG::GFP) or *S*. Typhi BRD948(pssaG::GFP). Cells were fixed and stained with DAPI plus antibody against common surface antigen and analysed by confocal microscopes.

**Fig 9:**
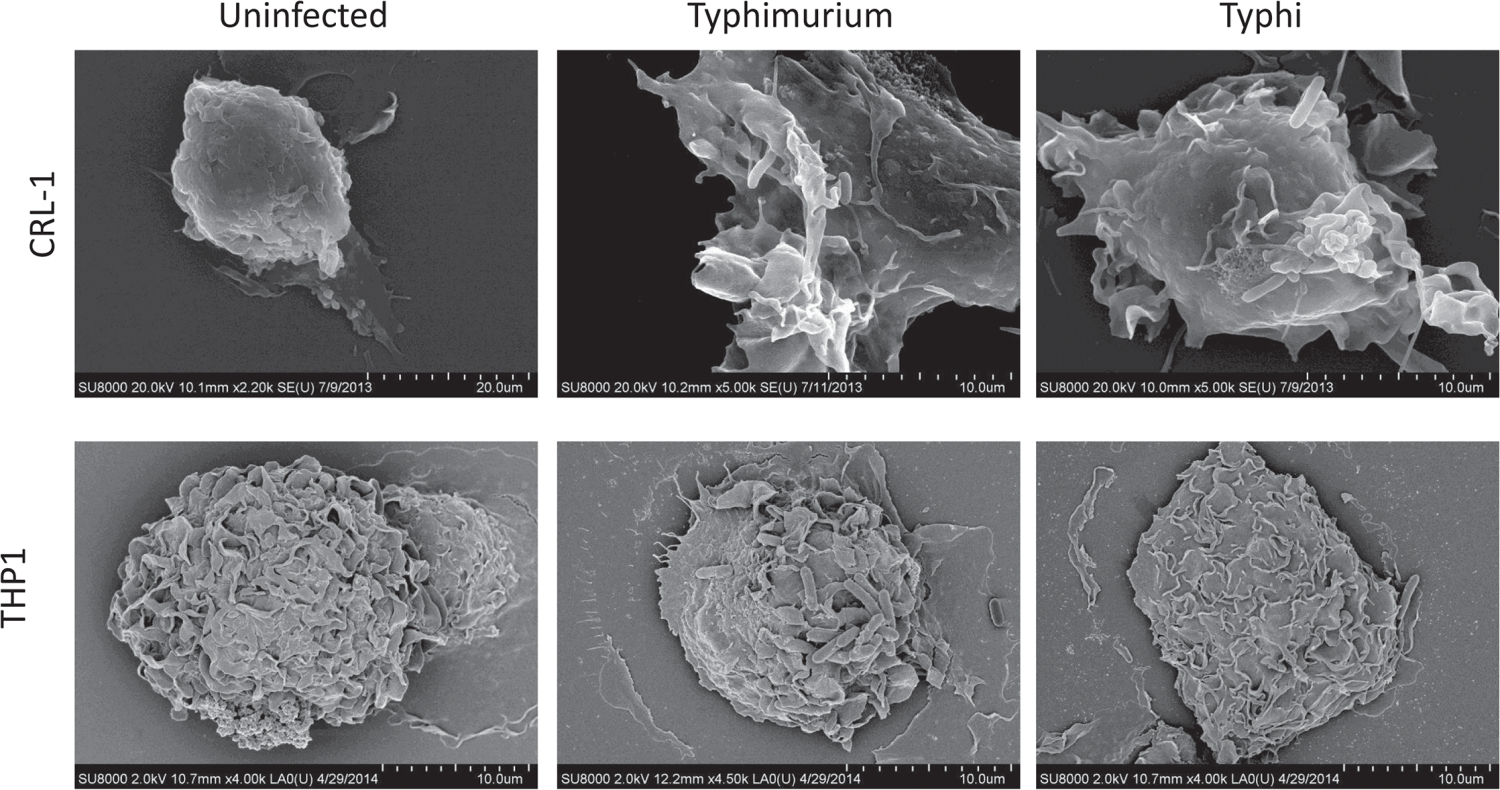
Scanning electron micrograph showing bacterial association and membrane ruffling in iPSDMs and THP-1 Mϕs. iPSDMs and THP-1 Mϕs were infected with *S*. Typhimurium SL1344 or *S*. Typhi BRD948 and cells were analysed by scanning electron microscopy.

Our data show both iPSDMs and THP-1 Mϕs efficiently took up *S*. Typhimurium while GFP and CSA expression was significantly co-localised indicating that the Salmonella were frequently residing within SCV’s **(** **Fig 8A** and **8B** **)**. By contrast, the levels of invasion by *S*. Typhi was more modest compared to *S*. Typhimurium in both Mϕ populations; being lowest in THP-1 **(Fig 8B)** compared to iPSDMs **(Fig 8A)**. This general lower level of invasion into Mϕs is a general feature of Vi-positive *S*. Typhi [23]. The initial interactions between Mϕ and these salmonellae were then investigated using scanning electron microscopy. Uninfected THP-1 Mϕs expressed some lamellipodia/phillopodia-like structures which were largely absent in iPSDMs in their basal state; similar structures have been observed following PMA treatment of THP1 cells. However, we observed that both *S* Typhimurium and *S* Typhi induced striking cytoskeletal changes and membrane ruffling in iPSDMs, although such morphological changes were less obvious in THP-1 Mϕ **(Fig 9**).

**Fig 10:**
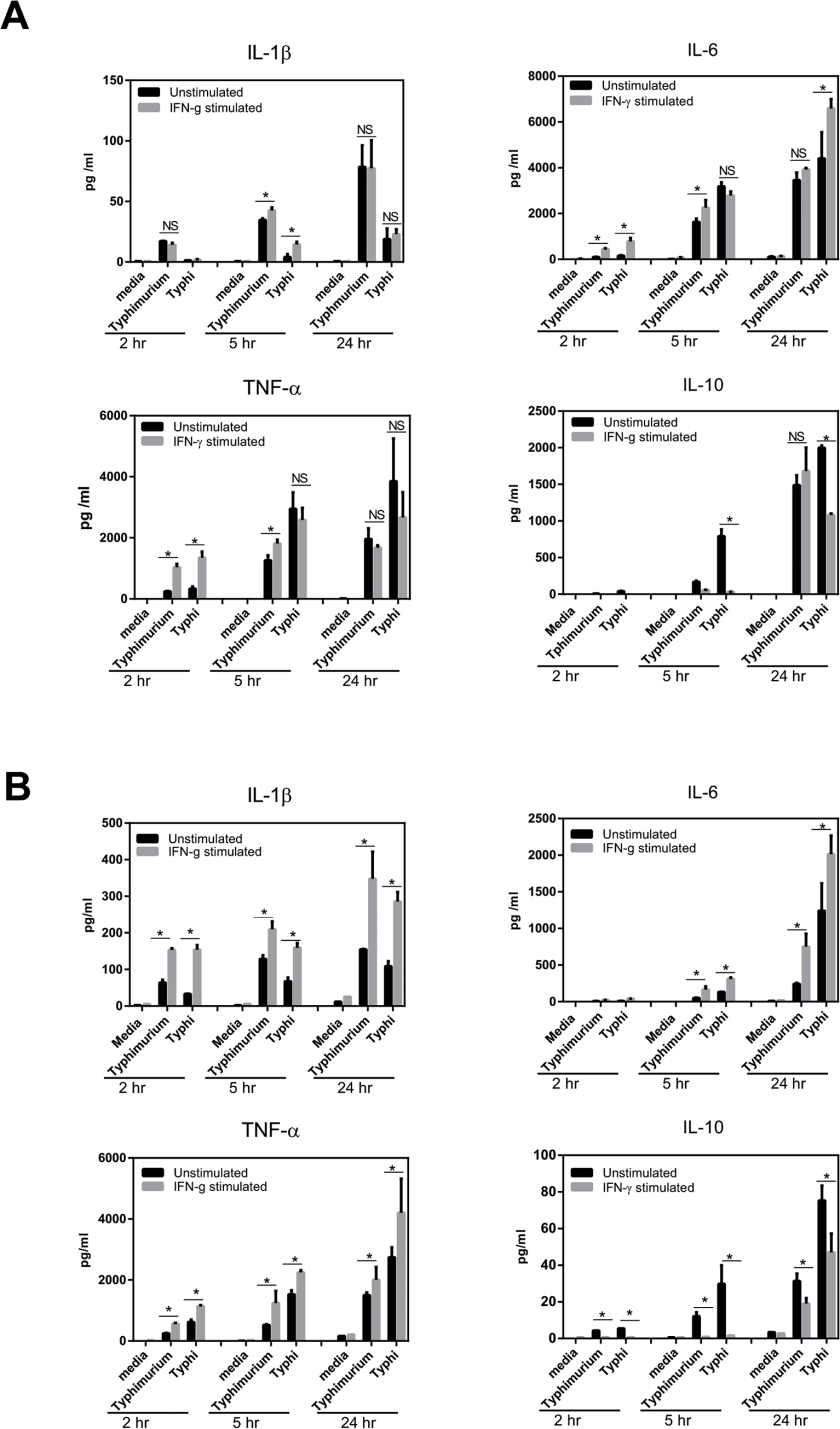
Activated iPSDMs show a stronger cytokine response compared to THP-1 Mϕs after Salmonella infection. Unstimulated and IFN-γ stimulated iPSDMs (A) and THP-1 Mϕ cell lines (B) were infected with *S*. Typhimurium SL1344(pssaG::GFP) or *S*. Typhi(pssaG::GFP) BRD948. Cytokine responses were measured 2, 5 and 24hr post infections by Millipore Milliplex custom kits on a Luminex FlexMap 3D. Representative data from 3 independent experiments are presented as mean ± SD. Statistical significance is compared between unstimulated and IFN-γ stimulated cells and presented as P<0.05(*), P<0.01(**) and non-significant (NS).

**Fig 11:**
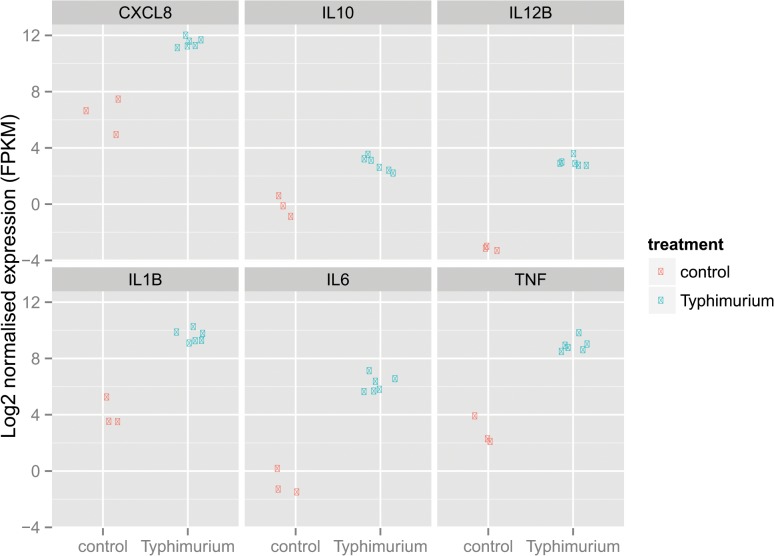
Salmonella induced cytokine response in iPSDM is transcriptionally regulated. Unstimulated and iPSDMs were infected with *S*. Typhimurium SL1344(pssaG::GFP) for 4 hours. RNA was extracted and sequenced from infected and uninfected iPSDMs and log2 normalised expression value for indicated cytokines were plotted. Data from three independent replicate is presented.

IFN-γ stimulated, classically activated Mϕs, can exhibit a robust cytokine response following pathogen exposure associated with efficient bacterial killing. Thus, we compared secretion of a range of inflammatory and immune-modulatory cytokines in response to *S*. Typhimurium and *S*. Typhi stimulation in IFN-γ pre-treated iPSDMs (**Fig 10A**) and THP-1 Mϕs (**Fig 10B**). Both iPSDMs and THP-1 Mϕs producedIL-1β, TNF-α and IL-6 and the relative levels were further enhanced in IFN-γ pre-treated Mϕs **(** **Fig 10A** and **10B**). Interestingly, cytokine levels were relatively higher in iPSDMs compared to THP-1 Mϕ cultures, with the exception of IL-1β, which was detected in higher levels in THP-1 Mϕ cultures. Furthermore, we observed that cultures from both cell types exposed to *S*. Typhi harboured high levels of IL-10 compared to *S*. Typhimurium **(** **Fig 10A** and **10B** **)**, potentially due to Vi expression [24]. Salmonella activate various pattern recognition and innate immune pathways and transcriptionally regulate inflammatory genes. We confirmed mRNA induction of various cytokine genes after Salmonella infection by RNASeq (**Fig 11**). Thus, our data show that innate immune and transcriptional machineries are functional in iPSDMs.

**Fig 12:**
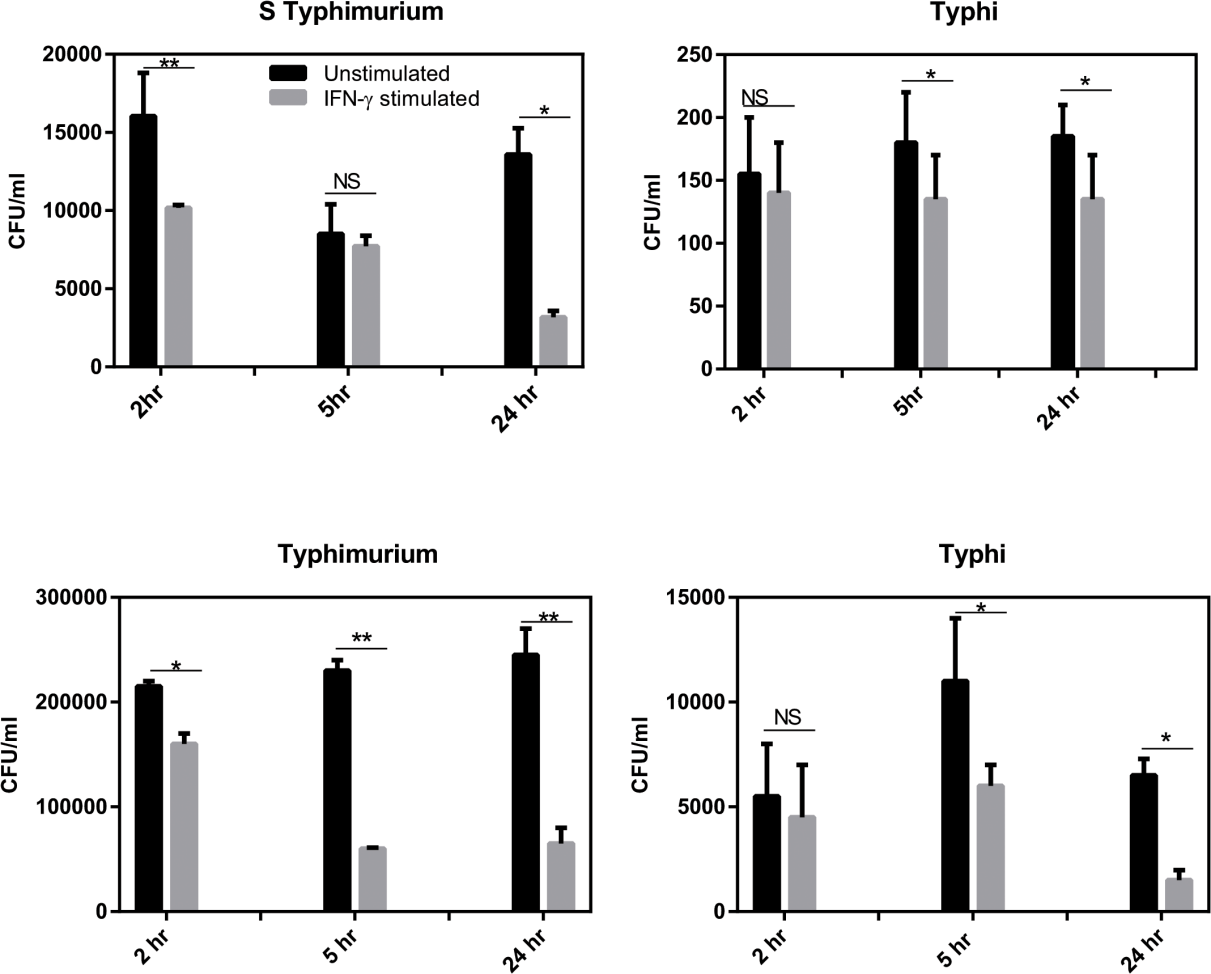
Activated iPSDMs show enhanced Salmonella killing compared to THP1 Mϕs. Unstimulated and IFN-γ primed iPSDMs (A) and THP-1 Mϕs (B) were infected with *S*. Typhimurium SL1344(pssaG::GFP) or *S*. Typhi(pssaG::GFP) BRD948. (MOI 10) and bacterial killing at different time points were compared by gentamicin protection assay and presented as CFU count. Representative data from 3 independent experiments are presented as mean ± SD. Statistical significance is compared between unstimulated and IFN-γ stimulated cells and presented as P<0.05(*), P<0.01(**),and non-significant (NS).

Finally, using a gentamicin protection assay, we compared killing of *S*. Typhimurium and *S*. Typhi in-IFN-γ stimulated iPSDMs and THP-1 Mϕs. IFN-γ pre-treatment enhanced killing of *S*. Typhimurium and *S*. Typhi in both Mϕ populations **(Fig 12)**. However, compared to THP-1(**Fig 12B),** Mϕs, iPSDMs **(Fig 12A)** showed more efficient killing of both Salmonella isolates further indicating that iPSDMs may be immunologically more active compared to THP-1 Mϕs. Furthermore, despite utilising the same MOI of infection, after the gentamicin protection assay lower numbers of viable *S*. Typhi were recovered from both Mϕs compared to *S*. Typhimurium **(Fig 12)**. Overall, our data suggest that iPSDMs efficiently take up Salmonella and generate a robust cytokine and bacterial killing response.

## Discussion

Here, we report a simple, efficient and reproducible method for generating large numbers of iPSDMs based on modifications of previously published differentiation protocols. The ability to generate high numbers of relatively homogeneous Mϕ cells with different genetic backgrounds and over extended periods should be of general value to the community working in this area of research. A number of factors emerged from this comparative study. The inhibitory effect of foetal calf serum on differentiation of myeloid precursors was striking. This could be due to high levels of latent TGF-β in foetal calf serum that are known to be a potent inhibitor of myeloid development [25]. The iPSDMs expressed established phenotypic markers of resting Mϕs and induced selective activation markers after classical and alternative activation. Furthermore, iPSDMs efficiently phagocytosed inactivated bacterial particles and produced inflammatory cytokines in response to specific TLR agonists. Cytokine response was generally more robust in iPSDMs compared to THP-1 Mϕs indicating the potential utility of iPSDMs in immunological studies. Direct comparison of iPSDMs and THP-1 Mϕs showed that both cells internalised and supported productive infections of *S*. Typhi and *S*. Typhimurium but activated iPSDMs exhibited a strikingly higher cytokine response and more efficiently killed these Salmonella compared to THP-1 Mϕs.

Although *S*. Typhimurium and *S*. Typhi are members of the same species, they show striking differences in host specificity and pathogenesis. Whilst *S*. Typhimurium infections are generally associated with self-limiting acute gastroenteritis in a broad range of hosts; *S*. Typhi is significantly host-restricted to human and is associated with the systemic disease typhoid. Interestingly, isolates representative of these two serovars induced different patterns of cytokines in both types of Mϕs, as well as different patterns of invasion and killing [26]. Genetic differences between these isolates are most likely responsible for their immunological and virulence phenotype. *S*. Typhi can express the Vi polysaccharide capsule, which has been shown to inhibit phagocytic uptake by Mϕs and modulate IL-10 production [23]. Thus, the iPSDM model described here can recapitulate several hallmarks of Salmonella interactions with Mϕs and also reflected subtle differences in cellular responses to these related serovars.

Host and pathogen factors that contribute to species restriction and intracellular adaptations of *S*. Typhi are poorly defined. Since pluripotent (ES and iPS) stem cells are particularly amenable to genetic manipulation, Mϕs derived from mutant iPSCs can be utilised to study specific gene function during infection; or genome-wide mutant libraries can be screened to discover new candidate genes involved in host defence against Salmonella and potentially other human adapted pathogens. Monocyte-derived Mϕs are the most accessible primary Mϕ population and are regarded as a gold standard in human Mϕ research, but these bone marrow-derived patrolling population are not true representatives of tissue resident mature Mϕs that originate from primitive haematopoietic precursors in early embryonic life and locally self-renew within adult tissues [27]. There is significant phenotypic and functional heterogeneity among tissue resident Mϕs and pathogens often show tropism for specific tissue resident Mϕ populations. Currently, options are limited to study pathogen interactions with tissue resident Mϕ populations. iPSC derived *in vitro* haematopoiesis resembles primitive haematopoiesis, and iPSDMs share phenotypic similarities with embryo-derived Mϕs [28]. In future, as our knowledge matures about specific signals and transcription factors that regulate development of tissue resident Mϕs, iPSDMs can be further reprogrammed into specific tissue resident populations. Some success is already achieved in developing selected tissue Mϕ populations [29,30].

In summary, iPSDMs are a flexible system to study host/pathogen interactions, especially human adapted pathogens. Furthermore, iPSDMs can provide a flexible and practical cellular platform for assessing host response in multiple genetic backgrounds. We also conclude that iPSDM approaches provide a novel genetically tractable and physiologically relevant cellular system that can be used to study fundamental macrophage biology beyond host pathogen interactions.
